# Emerging roles of ferroptosis in glioma

**DOI:** 10.3389/fonc.2022.993316

**Published:** 2022-08-22

**Authors:** Jiaqi Shi, Ning Yang, Mingzhi Han, Chen Qiu

**Affiliations:** ^1^ School of Medicine, Cheeloo College of Medicine, Shandong University, Jinan, China; ^2^ Department of Neurosurgery, Qilu Hospital, Cheeloo College of Medicine and Institute of Brain and Brain-Inspired Science, Shandong University, Jinan, China; ^3^ Jinan Microecological Biomedicine Shandong Laboratory and Shandong Key Laboratory of Brain Function Remodeling, Jinan, China; ^4^ Department of Epidemiology and Health Statistics, School of Public Health, Shandong University, Jinan, China; ^5^ Medical Integration and Practice Center, Cheeloo College of Medicine, Shandong University, Jinan, China; ^6^ Department of Radiation Oncology, Qilu Hospital, Cheeloo College of Medicine, Shandong University, Jinan, China

**Keywords:** ferroptosis, glioma, programmed cell death, iron metabolism, therapy resistance

## Abstract

Glioma is the most common primary malignant tumor in the central nervous system, and directly affects the quality of life and cognitive function of patients. Ferroptosis, is a new form of regulated cell death characterized by iron-dependent lipid peroxidation. Ferroptosis is mainly due to redox imbalance and involves multiple intracellular biology processes, such as iron metabolism, lipid metabolism, and antioxidants synthesis. Induction of ferroptosis could be a new target for glioma treatment, and ferroptosis-related processes are associated with chemoresistance and radioresistance in glioma. In the present review, we provide the characteristics, key regulators and pathways of ferroptosis and the crosstalk between ferroptosis and other programmed cell death in glioma, we also proposed the application and prospect of ferroptosis in the treatment of glioma.

## 1 Introduction

Ferroptosis is an iron-dependent regulated cell death driven by the peroxidation damage of phospholipid-containing polyunsaturated fatty acyl tails (PUFA-PLs) on the cell membrane or organelle membrane and subsequent membrane rupture (1). This non-apoptotic form of cell death triggered by erastin was first named in 2012 (2). Induced ferroptosis has been shown to be efficacious in eliminating drug-resistant tumor cells in various studies (3). Glioma is characterized by rapid proliferation and treatment resistance, and studies have demonstrated that inhibition of ferroptosis promotes malignant transformation, proliferation and angiogenesis in glioma (4, 5), so induction of ferroptosis is a promising research direction.

Herein, we summarize the processes of ferroptosis in glioma, the current findings on ferroptosis in glioma, which include some of the pivotal regulators and pathways relevant to ferroptosis and the crosstalk between ferroptosis and other programmed cell death including apoptosis, autophagic cell death, necroptosis and pyroptosis. Treatment resistance is an essential contributor to poor prognosis. Therefore, we focus on the relationship between ferroptosis-related metabolic processes and treatment resistance in glioma, and present the role of ferroptosis as well as its prospects in glioma treatment. The therapies include systemic therapy (especially temozolomide chemotherapy), radiotherapy, immunotherapy, and nanotherapy.

## 2 Regulation of ferroptosis in glioma

**Figure 1:**
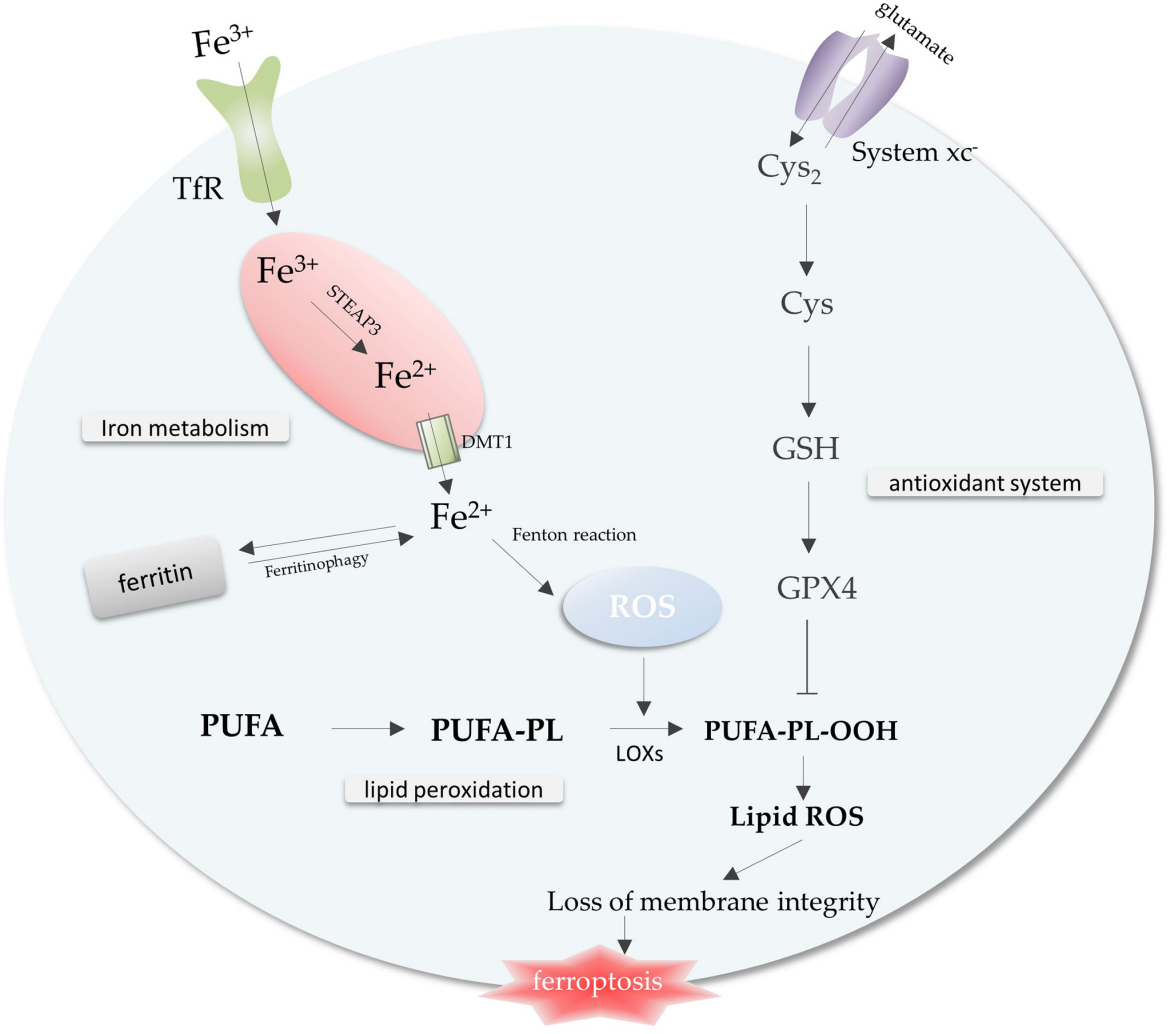
The core mechanisms related to ferroptosis. Free iron promotes intracellular ROS accumulation through the Fenton reaction, leading to lipid peroxidation and ferroptosis. The antioxidant system inhibits the lipid peroxidation process to prevent ferroptosis. Imbalance of iron metabolism, lipid peroxidation and antioxidant system leads to the occurrence of ferroptosis. Abbreviations: TfR, transferrin receptor; ROS, reactive oxygen species; Cys2, cystine; Cys, Cysteine; GSH, glutathione; GPX4, glutathione peroxidase 4; PUFA-PL, polyunsaturated fatty acid-containing phospholipid.

Iron metabolism, lipid peroxidation and antioxidant system, the imbalance among these three is a trigger for ferroptosis (**Figure 1**) . A complex regulatory network exists within the cell to regulate iron metabolism. Research indicates that free iron abundance promotes lipid peroxidation through the accumulation of reactive oxygen species (ROS) and the activation of iron-containing enzymes (6). Antioxidant systems in cells against ferroptosis mainly include Cysteine (Cys), glutathione (GSH), and glutathione peroxidase 4 (GPX4) axis (7) (**Figure 1**).

### 2.1 Iron metabolism

#### 2.1.1 Iron metabolism in ferroptosis

**Figure 2:**
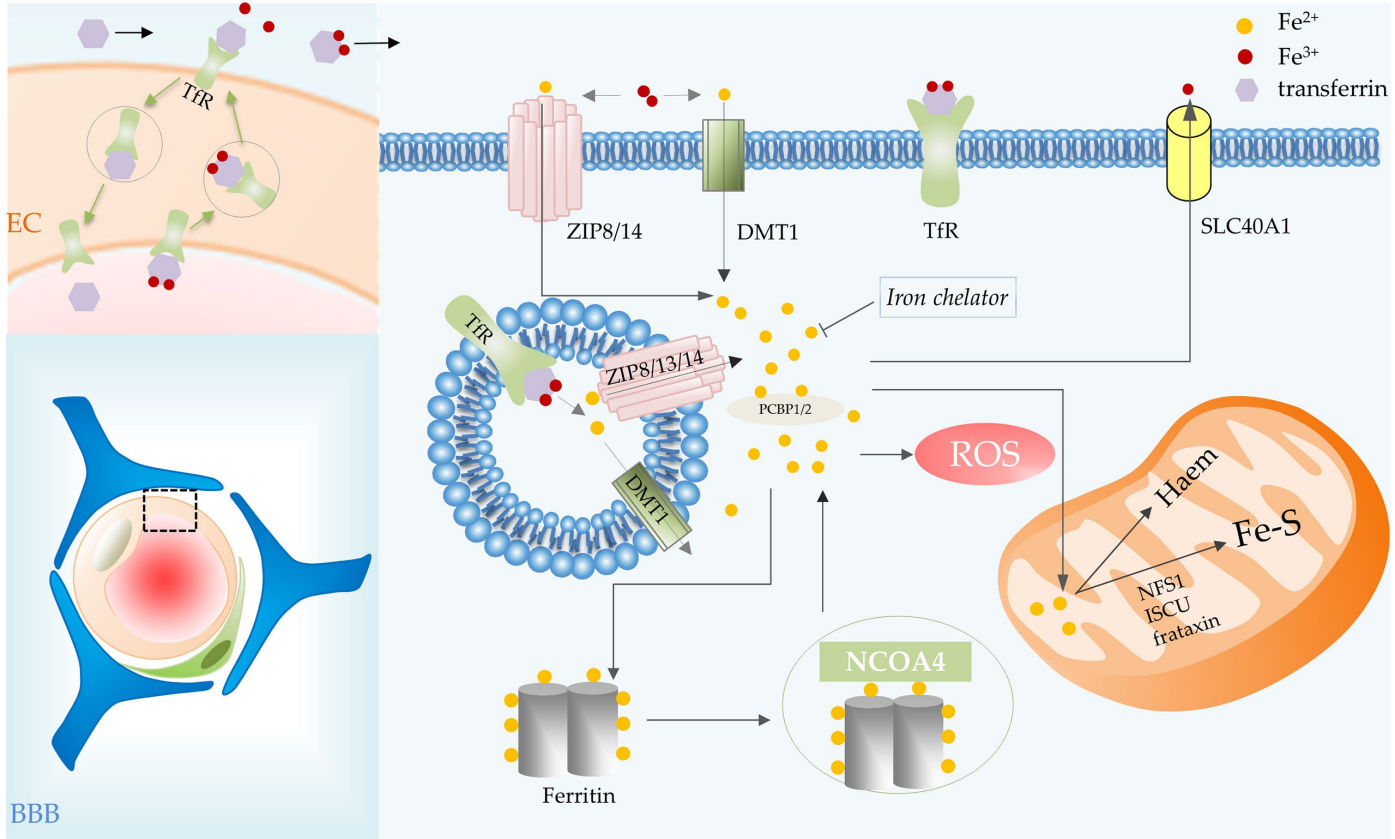
Iron metabolic processes involved in ferroptosis in glioma. BBB consists of ECs, basement membrane, pericytes, and astrocytic end feet. Iron transport depends on the TfR of vascular ECs in the BBB. Transferrin carries iron, the transferrin-receptor complex is internalized and then transported to the abluminal side of the endothelium. Transferrin combines with most of the iron that crosses the BBB, iron is then delivered to cells. In the cell, iron is released from transferrin in acidic endosomes. Endosomal iron can be delivered to the cytoplasm *via* DMT1, ZIP8, ZIP13 and ZIP14. Intracellular iron can be transported out of cells by SLC40A1, utilized by mitochondria for the synthesis of heme and Fe-S, and stored in ferritin. The iron in ferritin can be released by NCOA4-mediated ferritinophagy. Abbreviations: BBB, blood brain barrier; EC, endothelial cell; TfR, transferrin receptor; SLC40A1, solute carrier family 40 member 1; NCOA4, nuclear receptor coactivator 4.

Intracellular iron is strictly regulated. Most iron in cells is ligated by heme, stored in ferritin, an iron storage protein, or exists in the form of Fe-S clusters. Nevertheless, a small amount of labile iron is present in the cells and it is inclined to catalyze the formation of ROS (8, 9) (**Figure 2**). Iron mediates non-enzymatic peroxidation of lipid through Fenton reaction (8). The process of enzymatic peroxidation is iron-dependent because it requires the participation of iron-containing enzymes, such as ALOXs, NOXs, and CYP (10, 11).

Iron binds efficiently to extracellular transferrin, which has an important role in ferroptosis, and is released from transferrin when the iron is delivered to acidic endosomes *via* receptor-mediated endocytosis (10, 12). Transferrin transports iron into cells *via* transferrin receptors (TfR), TfR RNAi significantly inhibited cell death (12). Endosomal iron can be delivered to the cytoplasm *via* DMT1, ZIP8, ZIP13 and ZIP14 (13). Then PCBP1 delivers cytosolic iron to ferritin (an important iron storage protein in cells), non-heme iron enzymes and some other proteins (13, 14). PCBP2 is a DMT1-binding protein that transfers ferrous iron to the appropriate intracellular site or solute carrier family 40 member 1 (SLC40A1) (14). Nuclear receptor coactivator 4 (NCOA4)-mediated ferritinophagy is a form of selective autophagy that facilitates ferritin degradation leading to Fe^2+^ release (15). Transferrin and receptors promote ferroptosis by increasing intracellular iron content, whereas SLC40A1-mediated iron export inhibits ferroptosis (16). Additionally, ferroptosis is regulated by the iron-regulatory proteins (such as ACO1 and IREB2) at the translational level. Some mitochondrial proteins, such as cysteine desulfurase (NFS1), iron–sulfur cluster assembly (ISCU) and frataxin (17), restrain ferroptosis by increasing the synthesis of Fe-S clusters in cells (**Figure 2**).

#### 2.1.2 Iron transport in brain

Iron is an essential cofactor for many metabolic processes in the central nervous system (CNS), including DNA synthesis in neurons, oxidative phosphorylation, neurotransmitter production and oxygen transport (18, 19). However, brain is a very special organ in the human body, it is hidden behind the blood-brain barrier (BBB) with very low permeability, which limits its access to many substances (such as iron). Iron transport relies on the expression of TfR by vascular endothelial cells in the BBB. Transferrin binds to TfR expressed at the luminal membrane of endothelial cells. The transferrin-receptor complex will be internalized and then transported to the abluminal side of the endothelium. There, it will be exposed to the local microenvironment, which leads to the release of iron (18). Transferrin synthesized by oligodendrocytes combines with most of the iron that crosses the BBB after iron oxidation (20) (**Figure 2**).

#### 2.1.3 Iron metabolism in glioma

Compared to normal cells, tumor cells are more dependent on iron. In glioma, reprogrammed iron metabolism is regarded as a core factor in tumorigenesis, progression and the tumor microenvironment (21, 22). Transferrin receptor 1 (TfR1) controls the rate of iron uptake by glioma cells by regulating the amount of iron delivered to cells to meet metabolic requirements. Transferrin receptor 2 (TfR2) is frequently and highly expressed in glioblastoma (GBM) (23, 24). Immunohistochemistry of some GBM tissue samples with TfR mAbs exhibited a high rate of positivity (25). TfR2 expression in normal tissues is restricted, but a frequent expression of TfR2 on cancer lineages of distinct origins can be observed. It suggests that expression of TfR2 by tumor cells, along with increased expression of TfR1, may be a strategy for tumor cells to obtain optimal iron input (23).

Changes in transferrin and receptors can affect cellular iron content and may lead to the development of ferroptosis. Ferritin, composed of ferritin heavy chain (FTH1) and ferritin light chain (FTL), is an iron storage protein in cells. Recent findings strongly support the hypothesis that glial tumors synthesize and secrete ferritin (26, 27). Iron requirements are increased in glioblastoma stem cells, so high levels of cytoplasmic ferritin may protect cells from ferroptosis by enhancing iron chelation (28).

#### 2.1.4 Iron metabolism promotes glioma progression

Growing evidence suggests that iron metabolism-related processes in glioma cells contribute to tumor progression. GBM patients have elevated serum ferritin levels, probably due to the inflammatory state, and high serum ferritin levels are associated with poor prognosis (27). In glioma, high expression of TfR mediates intracellular iron accumulation and ROS formation, and promotes tumor proliferation. It also promotes an NMDA-receptor-mediated decrease in the number of neurons (29). Elimination of neurons is necessary for glioma cells to acquire space for growth. In addition, FTL is overexpressed in glioma (30). The oncogenic effect of FTL is mediated through the regulation of AKT/GSK3β/β-catenin signaling. It is proven that FTL promotes migration, invasion and chemoresistance in glioma (30).

#### 2.1.5 Iron metabolism and glioma treatment resistance

Notably, changes in iron metabolism are probably associated with the malignant transformation of glial cells and the degree of malignancy of glioma. The higher the grade of glioma, the correspondingly stronger the treatment resistance effect it has. Purified ferritin from glioblastoma-derived cell line has marked apoptosis-stimulating activity and are inhibited by neutralizing anti-ferritin antibody Ab rH02, however, isoferrin released from cultured neonatal astrocytes did not show this activity, suggesting that the transition of astrocytes to a malignant phenotype is accompanied by alterations in iron metabolic processes (26). And research demonstrated that the extent of TfR expression in glioma is positively correlated with tumor grading (29). FTL expression is also elevated in high-grade glioma (30). The main result of these changes is increased iron uptake by glioma cells. While iron may differentially affect the effectiveness of treatment by activating ROS production and/or signaling pathways including HIF-1 or IRP-1 recruitment (31).

In recent years, an increasing number of studies have found that the inevitability of treatment resistance and recurrence in GBM may be due to the presence of cancer stem-like cells (CSCs) (32). Owing to the nature of enhanced resistance to conventional chemo/radiotherapy and metastasis, CSCs have been proposed as promising targets for cancer eradication (33). Basuli et al. found that iron uptake by GBM CSCs is 2-3 folds higher than that of non-stem cell tumor cells (22). This alteration in iron metabolism is likely to be related to the treatment resistance of GBM CSCs. Expression of stemness-related markers has been proven to be affected by iron chelators, therefore targeting iron metabolic processes in CSCs is promising for reducing tumor cell therapeutic resistance (32).

### 2.2 Lipid metabolism

#### 2.2.1 Lipid metabolism in ferroptosis

In ferroptosis, the most essential process related to lipid metabolism is lipid peroxidation. Oxidized lipid disrupts and distorts the bilayer membrane. Oxidized lipid clusters in membranes destroy their barrier function by formation of hydrophilic pores, leading to ferroptosis (34).

**Figure 3:**
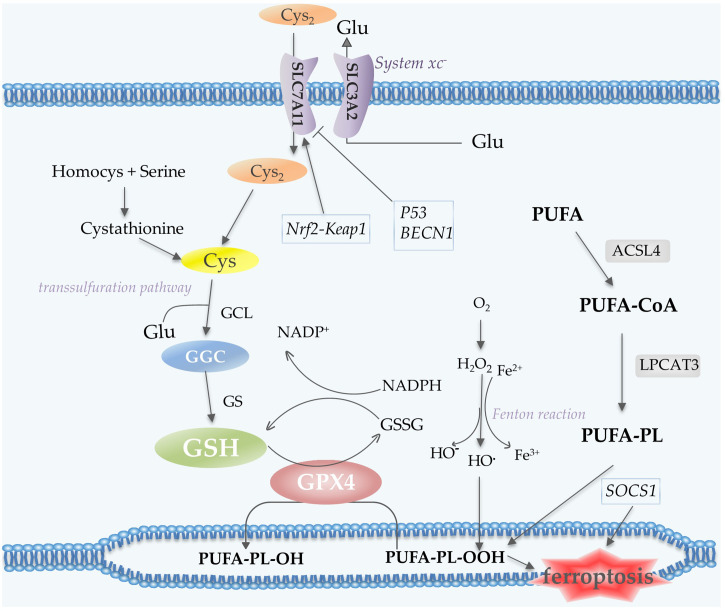
Lipid peroxidation and antioxidant processes in ferroptosis. System xc^-^ contains two subunits, SLC7A11 and SLC3A2, which mediate the transport of Cys_2_ and Glu. Cys_2_ is an ingredient for the synthesis of GSH, which together with GPX4 acts as the reductant for the inhibition of ferroptosis. Cys can also be generated *via* the transsulfuration pathway. PUFA undergoes a series of oxidation reactions and the product PUFA-PL-OOH leads to the occurrence of ferroptosis. Fenton reaction contributes to lipid peroxidation. P53 and BECN1 function as inhibitors of SLC7A11, while Nrf2-Keap1 pathway promotes the expression of this subunit. Abbreviations: Cys_2_, cystine; Glu, glutamate; GSH, glutathione; GPX4, glutathione peroxidase 4; Cys, Cysteine; PUFA-PL, polyunsaturated fatty acid- containing phospholipid; Nrf2, nuclear factor erythroid 2-related factor 2.

Polyunsaturated fatty acid (PUFA)-containing membranes are susceptible to oxidation (6, 13) (**Figure 3**). Long-chain fatty acid–CoA ligase 4 (ACSL4) has a preference for long-chain PUFAs such as arachidonic acid (AA) and adrenic acid (AdA). In an ATP-dependent manner, ACSL4 catalyzes the binding of AA or AdA to coenzyme A to form derived AA-CoA or AdA-CoA (35, 36), which are then esterified under the catalysis of LPCAT3 and form PUFA-PE by reaction with phosphatidylethanolamine (PE). Then lipoxygenases (LOXs) are required for ferroptosis to oxidize AA-PE and AdA-PE to hydroperoxides AA–PE-OOH and AdA–PE-OOH (13, 37). Six arachidonic acid lipoxygenase (ALOX) genes in humans (ALOX5, ALOX12, ALOX12B, ALOX15, ALOX15B and ALOXE3) have distinct expression profiles in different tissues (37). Other contributors of fatty acids include Acetyl-CoA carboxylase (ACC)-mediated fatty acid synthesis (38), and lipophagy-mediated fatty acid releasing (6).

#### 2.2.2 Lipid metabolism in glioma

Increased fatty acid synthesis and increased cholesterol uptake are considered to be features of malignant glioma. The altered lipid metabolism may mediate the resistance to chemotherapy and radiotherapy in GBM (39). Lipid peroxidation also contributes significantly to treatment resistance (40). Therefore, targeting glioma lipid regulation is one of the therapeutic strategies. The levels of PUFA are much higher in glioma than in normal brain tissue (41). However, glioma cells can appropriately reduce lipid peroxidation during lipid metabolism to avoid ferroptosis. It is notable that ACSL4 protein expression level was found to be decreased in glioma cells and BAO et al. found that knockdown of ACSL4 reduces ferroptosis and stimulates cell proliferation in glioma cells. In contrast, ACSL4 overexpression decreased the expression of GPX4, while upregulating the expression of ferroptosis indicators such as 5-HETE (5). Liu et al. reported a significant difference in the expression of ALOX5 in glioma and normal brain tissue (42). The above studies prove that many differences in lipid metabolism exist between glioma and normal tissues.

### 2.3 Cys, GSH, and GPX4 axis

#### 2.3.1 Cys, GSH, and GPX4 axis in ferroptosis

The Cys_2_/Glu antiporter system xc^-^, which is composed of two subunits SLC7A11 and SLC3A2, is needed to import cystine (Cys_2_) into cells for subsequent GSH synthesis (6). In the cell, Cys_2_ is oxidized to Cys, which is then synthesized with glutamate (Glu) by glutamate-cysteine ligase (GCL) to form GGC, and subsequently synthesize GSH catalyzed by glutathione synthetase (GS) (43). A cycle can be envisaged in which Glu can enter the cell *via* its transporter protein and be exported *via* the xc- reverse transport system, thus supporting the cellular uptake of Cys_2_ (44). Cys for GSH synthesis can also be obtained from protein degradation within the lysosome and transsulfuration pathway (44). Transsulfuration promotes the sulfur oxidation of homocysteine/methionine. Methionine is converted to homocysteine *via* the transsulfuration pathway, which is then converted to cystathionine and finally to Cys under the catalysis of cystathionine-γ-lyase (45, 46). GPX4 exhibits excellent resistance to irreversible peroxidation in the utilization of GSH to reduce hydrogen peroxide (H_2_O_2_) or organic hydroperoxides to water or the corresponding alcohols, while GSH is oxidized to glutathione disulfide (GSSG) (47, 48) (**Figure 3**).

#### 2.3.2 Cys, GSH, and GPX4 axis in glioma

System xc^-^ is vital in the survival of glioma cells. In glioblastoma cells, most of the Cys is derived from the reduction of Cys_2_ imported by the system xc^-^ (46). Cancer cells exhibit higher ROS levels compared to normal cells (49), and this leads to higher expression of NOXs and GPX4. Cancer cells can use GSH to reduce oxidation products and inhibit cell death, causing resistance to treatment (50). In GBM cells, high GSH/GPX4 levels induce epithelial-mesenchymal transition, leading to tumor progression, metastasis and chemoresistance (50).

## 3 Current status of ferroptosis studies in glioma

### 3.1 Regulators and pathways

#### 3.1.1 SLC7A11/xCT

Several studies have documented that glioma cells upregulate the expression of SLC7A11 (xCT). Regulation of SLC7A11 does not alter cell proliferation, but its overexpression increases the growth of anchorage-independent cells (51). Increased SLC7A11 expression correlates with tumor invasion and prognosis in patients with GBM. SLC7A11 is an independent predictive factor in GBM (52).

A number of studies have reported that the system xc^-^ is related to many properties of glioma cells. For example, system xc^-^ is the main pathway of the release of glutamate, glutamate excitotoxicity kills surrounding neurons, thus enhancing tumor invasion and growth. Glutamate release from glioma is also considered to be associated with tumor-associated seizures (53). High expression of SLC7A11 becomes an independent biomarker of seizures (54). Overexpression of SLC7A11 in anti-VEGF-treated GBM cells results in elevated extracellular glutamate. Glutamate promotes regulatory T (Treg) cells proliferation, activation, and suppressive function (55). This immunosuppression can be alleviated by reducing Treg cells to enhance the antitumor effect. In recent years, the relationship between SLC7A11 and cellular ferroptosis has also been elucidated. SLC7A11 promotes the absorption of Cys_2_, which in turn supports GSH synthesis and inhibits ferroptosis in tumor cells. This has also resulted in the birth of many drugs. In addition, although Cys_2_ uptake is essential for antioxidant protection of cancer cells against ferroptosis, Cys_2_ transport through SLC7A11 can also induce oxidative stress and cell death in glucose-deprived glioblastoma cells (56). Moreover, some research reported that cell survival under glucose deprivation conditions also depends on cell density. High cell density inactivates mTOR and promotes lysosomal degradation of SLC7A11, enhancing the viability of GBM cells under glucose-restricted conditions (57). While EGF contributes to cell death under glucose-deprived conditions by upregulating SLC7A11 at the transcriptional and protein levels (58). SLC7A11 overexpressing U251 glioma cells exhibit actin cytoskeletal changes reminiscent of epithelial-like cells and display an increased CSC-like phenotype, which might cause tumor drug resistance and recurrence (59).

#### 3.1.2 p53

The p53 gene has been identified as the most commonly mutated tumor suppressor gene in human cancers. It can transcriptionally regulate a range of genes to modulate DNA damage repair, cell cycle arrest, senescence, apoptosis and ferroptosis (60). Previous results showed that almost 50% of glioma samples have tumor protein p53 (TP53) mutations. This number is even higher when alterations in the p53 pathway are taken into consideration. The p53 gene or pathway is more frequently mutated in astrogliomas and GBM (60, 61). The regulatory network for p53 expression in glioma cells is very complex. The latest report has verified that p53 in glioma reduces MDM2 levels by inducing expression of miR-29a, thus reducing the degradation of p53 by MDM2, forming a feedback loop (62).

The role of p53 has a double-sided regulation mode in cells. p53, the first gene to be studied for increased susceptibility to ferroptosis, can inhibit the transcription of SLC7A11, leading to Cys deprivation. It was demonstrated that p53 downmodulates the level of histone H2B monoubiquitination (H2Bub1), which is involved in ferroptosis regulation by controlling the expression of the downstream gene SLC7A11 (63). P53 acts on glutaminase 2 (GLS2) to increase GSH hydrolysis, causing GXP4 inactivation. It also acts on lipid peroxide synthase to increase cellular susceptibility to ferroptosis (63, 64). SAT1, as a transcriptional target of p53, induces lipid peroxidation and allows cells to undergo ferroptosis in response to ROS-induced stress (65). It has been reported that TP53-induced glycolysis and apoptosis regulator (TIGAR) are direct targets of ferroptosis mediated by p53. TIGAR and cytochrome c oxidase 2 (SCO2) promote the pentose phosphate pathway (PPP) which is engaged in the production of NADPH, a reducing agent during ferroptosis. Additionally, PVT1 may mediate the role of p53 in promoting ferroptosis (63). MDM2 and MDMX, negative regulators of p53, normally promote ferroptosis (66). TP53 can prevent ferroptosis by inhibiting dipeptidyl-peptidase-4 (DPP4) activity and relevant findings have suggested that DPP4 is a key co-ordinator of lipid metabolism in colorectal cancer (67). Nucleotide synthesis is an essential cellular metabolic process, and a recent report indicates that the p53 pathway can inhibit the expression of ribonucleotide reductase, leading to GSH accumulation and the avoidance of ferroptosis (68). P53 could positively and negatively regulate ferroptosis (69), the role of p53 in ferroptosis needs to be further elucidated. Nevertheless, the role of p53 is influenced by several factors, such as cell type and p53 mutation. In normal tissues, wild-type p53 exhibits positive regulation of ferroptosis to prevent the accumulation of genetic mutations but inhibits ferroptosis to protect cells under stressed conditions (63, 70). In tumors, other ferroptosis regulators supersede the role of p53, and the effect of wild-type p53 appears to be limited. Conversely, mutant p53 renders tumor cells sensitive to ferroptosis (70). In summary, studies on the ferroptosis-related pathways involved in p53 in glioma and their distinction from other tumors are not numerous and deserve further study in the future.

#### 3.1.3 BECN1/Beclin1

BECN1, a haploinsufficient tumor suppressor gene (71), its dysfunction is correlated with many diseases, including carcinoma and neurodegeneration (72). BECN1 is a core autophagy protein essential for the autophagosome nucleation phase in mammals. ROS levels are higher in cancer cells compared to normal cells, and it is widely believed that high ROS levels induce autophagy. Recent studies have demonstrated that hTERT in GBM can reduce autophagy levels by inhibiting BECN1, leading to increased ROS and ultimately cell death (73). High expression of autophagy-related proteins such as BECN1 is more pronounced in high-grade glioma than in low-grade, so BECN1 might be a prognostic marker for glioma patients (74).

The role of BECN1 in ferroptosis has also received attention in recent years. Experimental data demonstrated that BECN1 does not influence intracellular iron accumulation or expression related to iron metabolism (75). BECN1 promotes ferroptosis by the direct blockade of the SLC7A11 subunit of system xc^-^. AMPK-mediated phosphorylation of the BECN1 Ser90/93/96 sites is essential for BECN1 to form a complex with SLC7A11 (76).

#### 3.1.4 SOCS1

Suppressor of cytokine signaling 1 (SOCS1) has been demonstrated to be a tumor suppressor capable of bridging p53 and ATM at sites of DNA damage, leading to p53 phosphorylation and consequently increasing its transcriptional activity (77). It has been proven that the expression of SOCS1 decreases the levels of SLC7A11 and GSH in cells, suggesting its ability to sensitize cells to ferroptosis (78). Ferroptosis-related gene SOCS1, has become a biomarker for the diagnosis or prognosis of many diseases, such as tuberculosis (79), AML (80) and head and neck squamous cell carcinoma (HNSCC) (81).

The effects of SOCS proteins in GBM have recently become a research hotspot. SOCS inhibits proliferation and angiogenesis of GBM through the negative regulation of the JAK/STAT3 signaling pathway and SOCS proteins can also control the invasion and metastasis of GBM through multiple pathways (82). Evidence indicated that SOCS1 and SOCS3 might be involved in tumor aggressiveness and radiation tolerance (83). It has been implicated that SOCS1 tends to be repressed in GBM as a result of CpG island-mediated epigenetic silencing of the SOCS1 locus. Reintroduction of SOCS1 can sensitize cells to radiation (84). Mutation status of p53 may have a regulatory role in the transcriptional plasticity of the SOCS1 promoter (85). Increasing SOCS1 expression appears to simultaneously induce ferroptosis and improve radiotherapy sensitivity, and SOCS1 is a possible therapeutic target for glioma. However, SOCS1 is involved in a complex regulatory network, and the role of SOCS1 in inducing ferroptosis in glioma cells is currently less known.

#### 3.1.5 Frataxin

Frataxin, a highly conserved protein, is localized in the mitochondrial matrix and is involved in the biosynthesis of Fe-S clusters. Frataxin is a key regulator of ferroptosis *via* the regulation of iron homeostasis and mitochondrial function (86). Frataxin can accelerate the rate of persulfide formation on NFS1, promoting Fe-S cluster synthesis (87). Suppression of frataxin significantly inhibits proliferation, disrupts mitochondrial morphology, blocks Fe-S cluster assembly and exacerbates iron accumulation (86). Insufficient maintenance of Fe-S clusters strongly activates the iron starvation response and combines with inhibition of GSH biosynthesis (88). It has been shown that frataxin has a tumor suppressive effect, but it has a dual, pro-proliferative role in astrocytic tumors (89).

#### 3.1.6 Nrf2-Keap1 pathway

Nuclear factor erythroid 2-related factor 2 (Nrf2) is a critical transcription factor in the cellular response to oxidative stress. Nrf2-dependent transcription is repressed by Keap1 under basal conditions, when cells are exposed to oxidative stress, Nrf2 escapes repression and activates antioxidant responsive element (ARE)-dependent gene expression (90). Nrf2 is overexpressed in GBM cells and associated with poor prognosis (91). Nrf2-Keap1 pathway is involved in ferroptosis in glioma. Activation of Nrf2-Keap1 signaling can upregulate SLC7A11. It was also found that fostering Nrf2 expression and inhibiting Keap1 both increase the resistance to ferroptosis in glioma cells.

#### 3.1.7 COPZ1/NCOA4/FTH1

COPZ1 expression is upregulated in GBM cell lines and it has a tendency to negatively regulate the activity of NCOA4. Knockdown of COPZ1 leads to an increase of NCOA4, contributing to the degradation of ferritin, which leads to increased intracellular ferrous iron levels and eventually to ferroptosis. Thus, the COPZ1/NCOA4/FTH1 axis is a novel therapeutic target for the treatment of GBM (92). Targeting pathways including NCOA4 may be a promising approach for the treatment of glioma.

### 3.2 Crosstalk between ferroptosis, apoptosis, autophagic cell death, necroptosis and pyroptosis in glioma

Apoptosis, autophagic cell death, necroptosis, pyroptosis and ferroptosis represent a group of highly ordered programmed cell death (PCD) events that can eliminate cells that are running chaotically or destined to die. In tumor cells, survival signaling and programmed death resistance (such as apoptosis resistance) are two complementary aspects. Targeting increased survival may not be effective without also addressing cellular PCD resistance (93, 94). Therefore, it is especially necessary to explain the process of PCD and the crosstalk between them, which might provide new strategies for tumor treatment.

#### 3.2.1 Apoptosis

**Figure 4:**
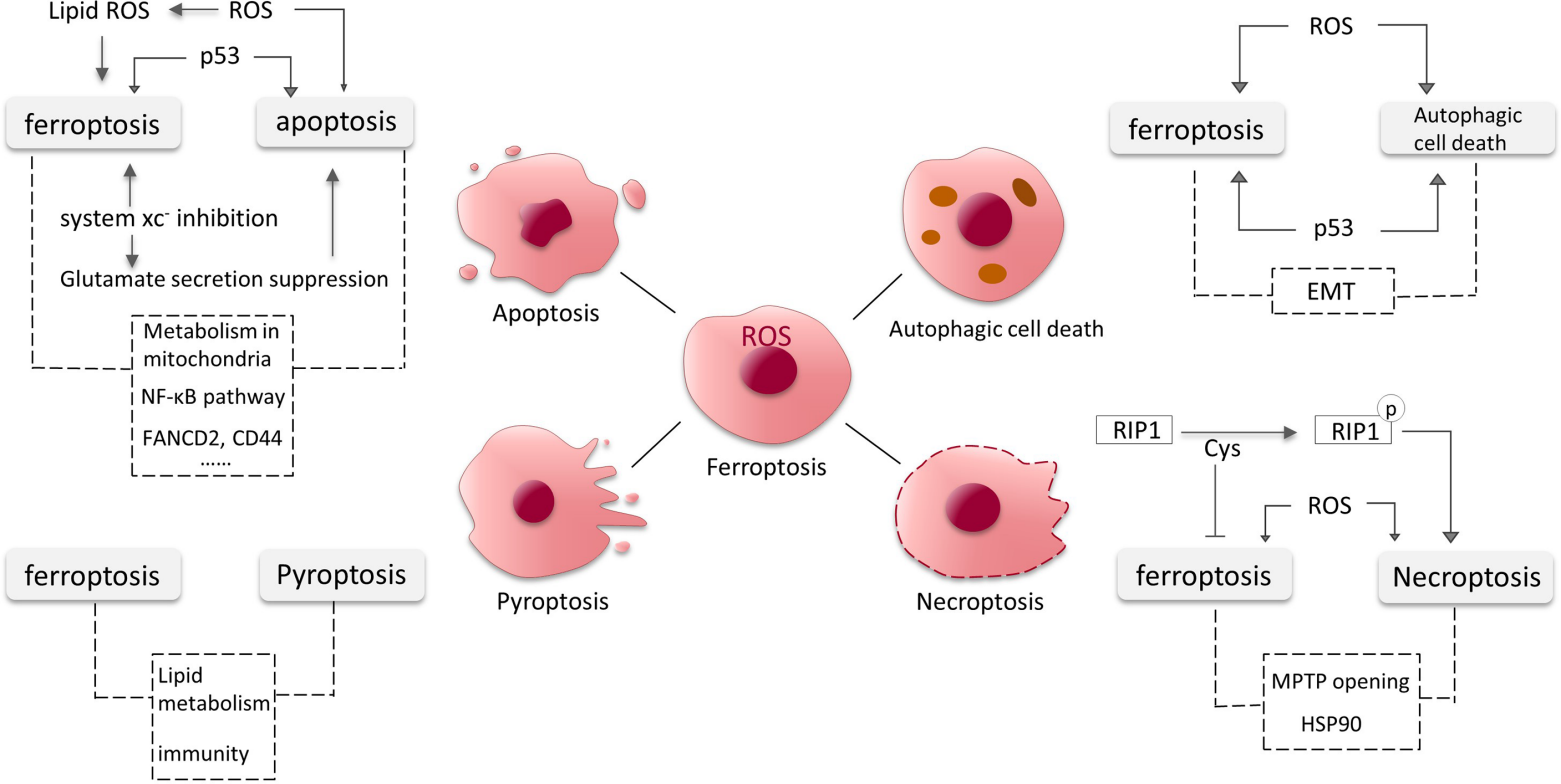
Crosstalk between ferroptosis, apoptosis, autophagic cell death, necroptosis and pyroptosis in glioma. The dotted lines represent possible intersections and require more research to elucidate.

Apoptosis is a relatively early discovery and well-studied PCD, and here we will present some findings about the intersections of ferroptosis and apoptosis in glioma (**Figure 4**).

In GBM cells, ROS controls cellular stability by affecting different signaling pathways. It is a pivotal participant in the occurrence of ferroptosis. It has been found that excess ROS can also induce apoptosis (95). ROS may act as a key substance in the onset of apoptosis and ferroptosis in GBM cells. As a tumor suppressor molecule, p53 increases ferroptosis susceptibility and can also induce cell cycle arrest and apoptosis. Dysregulated p53 pathway is relevant to apoptosis evasion (96). We have previously mentioned that system xc^-^ mediates the toxic secretion of most glutamate from GBM cells, and its inhibition induces ferroptosis. A recent study revealed that the suppression of extracellular glutamate release promotes apoptosis and autophagy in GBM cells (97). Moreover, RSL3 (a GPX4 inhibitor) was found to drive ferroptosis *via* NF-κB pathway in GBM cells (98), and NF-κB pathway is also involved in apoptosis (99, 100). Down-regulation of FANCD2 and CD44 expression by sponging hsa-miR-27a-3p promotes apoptosis and ferroptosis in glioma cells, the pathways involved might also be intersections of apoptosis and ferroptosis (101). Mitochondria are known to be involved in various PCD processes including apoptosis, and the role of mitochondria in ferroptosis is gradually being discovered and the connections deserve further studies (102, 103).

#### 3.2.2 Autophagic cell death

Autophagic cell death, independent of caspase, can be defined as cell demise with strict requirements for autophagy (104, 105). In tumors, autophagy has both pro-survival and pro-death functions (106). Nevertheless, autophagic cell death exhibits extensive autophagic degradation (104). GBM cells have lower levels of autophagy-related proteins compared to low-grade astrocytomas (107). Autophagy is an attractive target for anti-cancer therapy (108).

Similar to apoptosis, p53 and excess ROS also induce autophagy in GBM cells (95, 107). NF-κB is released from the BNIP3 promoter and permits the action of E2F1 to induce autophagy under hypoxic conditions (107). Epithelial mesenchymal transition (EMT) promotes aggressive migration, immunosuppression and drug/radiotherapy resistance of cancer cells (109). EMT processes might also be linked to both autophagy and ferroptosis in glioma. An increasing number of studies have shown links between ferroptosis and EMT in tumor cells. For instance, increased levels of H_2_O_2_ associated with EMT confers susceptibility to ferroptosis (110). The crosstalk between autophagy and EMT processes is complex. In the early stages of metastasis, autophagy primarily inhibits the EMT programme, and later, metastatic cells may require sustained autophagy for survival under environmental and metabolic stress conditions (111) (**Figure 4**).

#### 3.2.3 Necroptosis

Necroptosis is a newly found PCD that combines necrosis and apoptosis. It is regulated by caspase-independent pathway and has morphological characteristics of necrosis (112, 113). Necroptosis is regulated by receptor-interacting protein (RIP) 1 activation (114).

Cys plays important roles in ferroptosis and necroptosis. The three cysteines in RIP1 form intermolecular disulfide bonds, which induce ROS generation and consequently RIP1 autophosphorylation, promoting necroptosis (114). Necroptosis can also be induced by high levels of intracellular ROS (115). Meanwhile, Cys is involved in the synthesis of GSH to inhibit ferroptosis. Mitochondrial permeability transition pore (MPTP) opening and heat shock protein 90 (HSP90) also could be the intersections between ferroptosis and necroptosis (114) (**Figure 4**).

#### 3.2.4 Pyroptosis

Pyroptosis is a novel PCD mediated by gasdermin D protein and triggered by certain inflammasomes (116, 117). Pyroptosis can affect tumor proliferation, invasion and metastasis (117). Recent studies have revealed its role in gastrointestinal cancer, hepatocellular carcinoma, breast cancer and other cancers (118–121). Pyroptosis is a vital regulator of the immune microenvironment and a prognostic predictor in glioma (122), and relevant studies are currently limited.

Lipid also seems to be associated with pyroptosis induction. Substantial lipid aggregation induces activation of pyroptosis signaling pathways in the formation of vulnerable atherosclerotic plaques (123). Lipid peroxidation can drive pyroptosis in lethal polymicrobial sepsis (124). Lipid levels are elevated in glioma cells and the metabolism of lipid is critical in ferroptosis, but it is unclear whether lipid in glioma contributes to pyroptosis. Nitric oxide (NO) is involved in cell proliferation, cardiovascular formation and apoptosis in glioma (125, 126). Many recent studies have indicated that inhibition of NO mediates the processes of ferroptosis and pyroptosis (127). It has also been mentioned that CD8^+^ T cells can suppress tumor growth by triggering ferroptosis and pyroptosis (128). The intersections and shared pathways of ferroptosis and pyroptosis in glioma require more research to be elucidated (**Figure 4**).

## 4 Ferroptosis in therapy of glioma

The applications of ferroptosis in the treatment of glioma are promising, and the induction of ferroptosis or ferroptosis inducers in combination with other treatments have proven to be effective in a variety of glioma cell lines, tissues, and animal models (**Table 1**), but the clinical applications currently have yet to advance.

**Table 1:** The promising applications of ferroptosis in the treatment of glioma.

Therapy	Type	Mechanism	Reference
Sulfasalazine	Glioma	SLC7A11 inhibition	(129)
Sorafenib	Glioma	SLC7A11 inhibition	(129)
Silibinin	Glioma	Silibinin downregulates SLC7A11 and depletes Cys in a time-dependent manner	(130)
TMZ + Erastin	Glioma	Erastin sensitizes tumor cells to TMZ by blocking SLC7A11 and reducing cystathionine-γ-lyase function	(129)
TMZ + ALZ003	GBM	ALZ003 sensitizes GBM to TMZ by inhibiting GPX4	(131)
TMZ + Sulfasalazine	GBM	Sulfasalazine enhances the cytotoxicity of TMZ	(132)
Lapatinib + Siramesine	Glioma	Inducing ferroptosis by increasing iron level	(133)
Microbeam irradiation + IKE/RSL3/sorafinib	GBM	Enhancement of cytoplasmic lipid peroxidation	(134)
Gamma knife radiosurgery + Sulfasalazine	GBM	Sulfasalazine inhibits SLC7A11 and promotes ferroptosis	(135)

TMZ, Temozolomide; IKE, Imidazole ketone erastin.

### 4.1 Therapeutic resistance in glioma

Glioma comprises 40% of all primary brain tumors and is a serious threat to human life. In particular, GBM is the most common and aggressive primary CNS malignancy, with a median survival of only 15 months despite various treatments including surgery, temozolomide (TMZ) chemotherapy, and radiotherapy (136, 137). The resistance of tumor cells to these therapies is an important reason for the poor prognosis.

The drivers of chemoresistance in glioma can be simply attributed to the influence of genetic aspects and the effects of the external environment. Altered expression of multidrug resistance (MDR)-related genes correlates with reduced treatment responsiveness (138). It has been observed that glioma can exhibit overexpression of ABC transporter proteins, which decrease therapeutic drug accumulation in tumor cells and are directly related to the chemoresistance (139). Further, this resistance is also associated with the DNA damage response of tumor cells (140), the mismatch repair system (141, 142), MGMT status (143, 144), and the regulation of a large number of microRNAs which involves a complex regulatory network consisting of various intracellular molecular signaling pathways (145–150). Tumor microenvironment containing endothelial cells, immune cells, stromal cells, noncellular factors and special conditions, also supports chemoresistance of tumor cells especially CSCs (140, 151). BBB prevents almost all large molecules and more than 95% of small molecules from entering the brain, resulting in unsatisfactory chemotherapy for glioma (152).

With some similarities to chemoresistance, the tumor microenvironment also plays an important role in radioresistance, and multiple signaling pathways (AKT pathway, notch pathway, Wnt/β-catenin pathway, STAT3 pathway and other pathways), proteins and microRNAs in differentiated glioma cells or CSCs have been shown to affect radiation resistance (153). Tumor cell networks with high cell density also have a significant resistance function. Research has revealed that the perivascular niche (the preferential location of quiescent glioma cells) and the formation of a multicellular network by tumor microtubules are involved in radiotherapy and chemotherapy resistance (154). Heterogeneity within GBM (regional genetic variance and cellular hierarchies often regulated by different CSC niches) is accepted as the basis for resistance to multiple treatments (155, 156). Moreover, the close association between hypoxia and resistance to radiotherapy in glioma, especially in GBM, deserves attention. Severe hypoxia is more common in GBM than in lower-grade glioma (157). Oxygenation is essential to the effectiveness of radiotherapy. Hypoxia also stimulates enzymes responsible for cancer survival under hypoxic stress *via* upregulation of HIF (158).

GBM is highly immunosuppressive and has multiple immune evasion mechanisms (159). Due to the special structure of the brain such as BBB, its immune environment is unique (160, 161). In brain tumor patients, immunological dysfunction is a major obstacle to immunotherapy (155). Some infiltrating immune cells in the tumor microenvironment such as Treg cells (162), myeloid-derived suppressor cells (163), etc. are also engaged in immunotherapy resistance. And the molecular heterogeneity of GBM hinders efforts to identify high-quality clonal neoantigens (155). In addition, glioma cells have potent adaptive and acquired resistance mechanisms, which involve genetic alterations shaped by immunological pressure (164). Probably the use of combined immune checkpoint blockade to overcome adaptive resistance is one solution (165).

Since therapeutic resistance is a major impediment to glioma treatment, we will focus on the relationship between ferroptosis and treatment resistance in various therapeutic approaches in the following introduction. In addition, we will also present the role and potential applications of ferroptosis in the treatment of glioma.

### 4.2 Systemic therapy

Chemotherapy and targeted therapy are critical aspects of malignancy treatment. In glioma, TMZ remains the mainstay of chemotherapy (166). However, drug resistance in glioma cells is currently a major challenge. A growing number of studies suggested that ferroptosis may be related to this resistance and that the efficacy of many drugs might also be associated with the ferroptosis induction.

#### 4.2.1 Temozolomide

TMZ was approved by the US Food and Drug Administration (FDA) for the treatment of adult refractory anaplastic astrocytoma in 1999 and newly diagnosed glioblastoma in adults in 2005 (167). TMZ, the most effective drug for the treatment of glioma, has the advantages of oral administration, easy penetration of BBB, and acidic environment stability. DNA methylation is regarded as the principal mechanism of cytotoxicity of TMZ to malignant cells (168). But its clinical efficacy is not ideal and glioma resistance to TMZ is the most important reason for chemotherapy failure. Ferroptosis has been shown to be linked to drug resistance of TMZ, and clarifying this relationship facilitates the application of ferroptosis to the clinical practice of glioma treatment.

**Figure 5:**
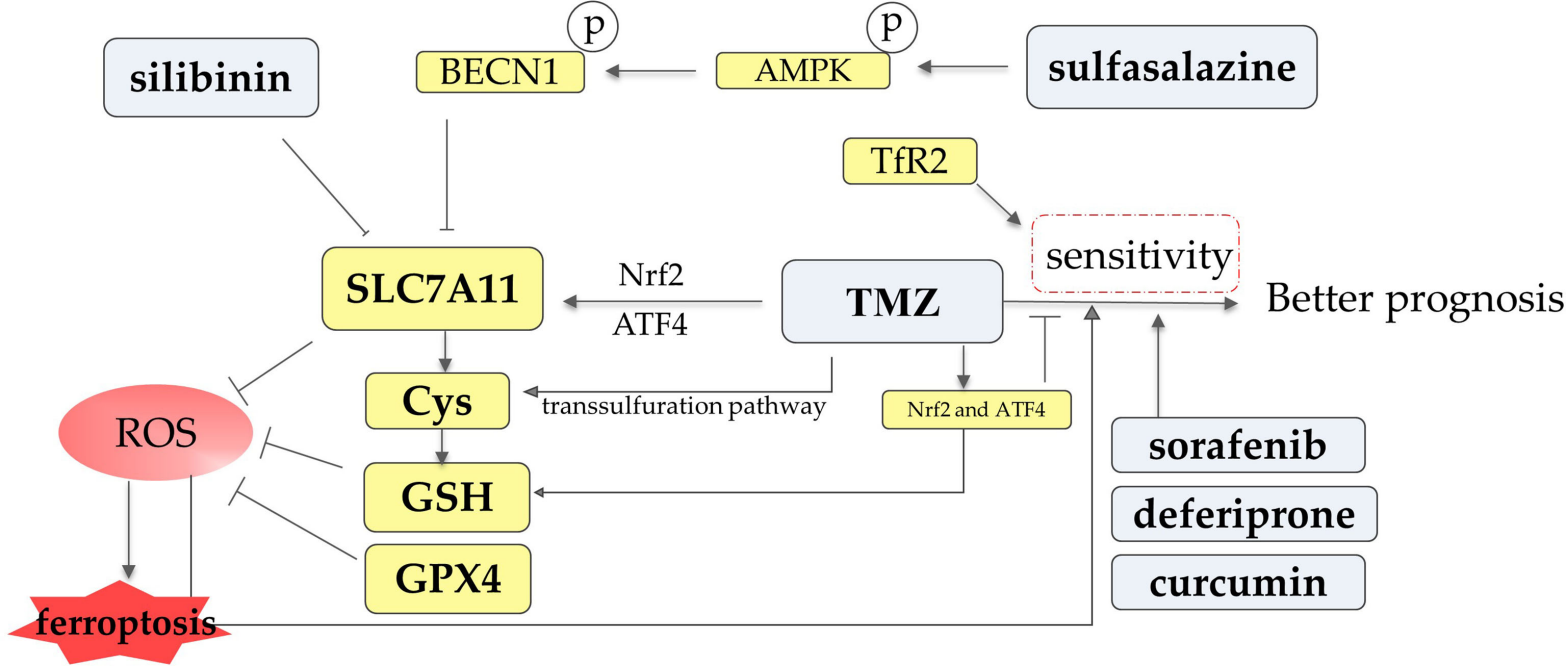
The association between drugs such as temozolomide (TMZ) and ferroptosis in glioma cells and some factors influencing TMZ sensitivity. TMZ increases the levels of Nrf2 and ATF4 and thus induces the expression of SLC7A11 and GSH *via* multiple mechanisms. Silibinin and sulfasalazine inhibit the SLC7A11 subunit. TMZ facilitates Cys synthesis through the transsulfuration pathway. SLC7A11, GSH, and GPX4 suppress ROS formation, while ROS promotes ferroptosis. ROS overproduction is likely to be important in enhancing TMZ sensitivity. Sorafenib, deferiprone, and curcumin also increase the sensitivity of glioma cells to TMZ.

Decreased GSH levels and GPX4 levels and inhibition of SLC7A11 can induce ferroptosis through the production of excess ROS. Chen et al. demonstrated that down-regulation of GSH levels could sensitize GBM cells to TMZ. TMZ significantly induces the expression of Nrf2 and ATF4 (**Figure 5**). Transcription factor Nrf2 can mediate TMZ resistance *via* the synthesis and utilization of GSH, and inhibition of Nrf2 increases the TMZ sensitivity of glioma cells (169, 170). ATF4 promotes the expression of GSH and SLC7A11 to avoid ferroptosis in glioma cells, inhibition of ATF4 can reduce the resistance of glioma cells to TMZ (171–173). SLC7A11 expression is enhanced by TMZ *via* Nrf2 and ATF4 activation pathway, and erastin-inhibited SLC7A11 enhances TMZ toxicity (173). It has also been observed that anti-treatment cells are GPX4-dependent and that loss of GPX4 function causes ferroptosis (174). Cystathionine γ-lyase (an enzyme involved in the transsulfuration pathway) is induced by TMZ to increase the supply of Cys (175). Overproduction of ROS is likely to be important in enhancing TMZ sensitivity (**Figure 5**), sorafenib alters TMZ sensitivity *via* autophagy and the JAK2/STAT3-AIF axis, and this alteration can be reversed by ROS clearance (176). It was also reported that the effectiveness of TMZ treatment is related to p53 status (177).

Some agents that function as iron chelators to suppress ferroptosis have been demonstrated to be associated with the reversal of TMZ resistance. Deferiprone (also known as ferriprox) is an orally active, brain-permeable drug. TMZ and deferiprone combination therapy significantly reduces cell viability in glioma cells (178). Curcumin, a component of the Indian spice turmeric, is able to sensitize GBM cells to TMZ treatment. The effect is achieved by enhancing apoptosis. The combination treatment of curcumin and TMZ was observed to have a synergistic effect in generating ROS, which may contribute to therapeutic sensitization (131, 179).

TMZ resistance is also associated with iron metabolic processes in glioma cells. In GBM patients treated with radiotherapy and temozolomide, a highly significant correlation was found between the level of TfR2 and overall survival (OS). One of the reasons is that TfR2-positive cells are more sensitive to TMZ (180, 181). Fluorescence density of PAMAM-PEG-Tf/TMZ in TfR+ glioma stem cells (GSCs) was significantly higher than that of matched non-stem cells and active apoptosis of tumor cells could be observed after the uptake of PAMAM-PEG-Tf/TMZ, suggesting that targeting transferrin receptors to deliver TMZ is a potential GSC-mediated treatment method (182).

#### 4.2.2 Sulfasalazine

Sulfasalazine is a drug widely used to treat a number of chronic inflammatory conditions (183). It is also an established inhibitor of system xc^-^ (**Figure 5**). Sulfasalazine impacts on ferroptotic cell death of tumors and has also been proven to alleviate glioma-related brain edema and epileptic events (129, 183).

Sulfasalazine did not show significant benefit in a small, discontinued phase I study, but it was not concluded to be ineffective given the patients’ health status, etc. Sulfasalazine might be used as an adjuvant treatment for malignant glioma (183). The combination of TMZ and sulfasalazine was shown to be cytotoxic to T98G and A172 cells, and sulfasalazine was found to enhance the cytotoxicity of TMZ to human GBM cells (132). Sulfasalazine and valproic acid drive GBM cell death through an imbalance in the intracellular oxidative response, making this drug combination a hopeful therapeutic strategy (184).

Many derivatives of sulfasalazine have been synthesized, and further studies on the molecular structure of system xc^-^ and its combination mode with inhibitors may help guide the design of potential inhibitors. SLC7A11 ligand models can be further optimized to find powerful lead molecules for the discovery of new drugs (185).

#### 4.2.3 Silibinin

Silibinin has been shown to be effective in removing tumor cells from breast cancer, colorectal cancer, glioma, etc. Silibinin is believed to result in glioma cell death through the induction of lethal autophagy, which is through the induction of oxidative stress-mediated BNIP3-dependent AIF nuclear translocation (186). Recent studies have found that silibinin leads to downregulation of SLC7A11 and also depletes Cys in a time-dependent manner, resulting in depletion of GSH and accumulation of ROS. BNIP3 plays an essential role in the functional performance of silibinin. Reduction of ROS with the antioxidant GSH significantly prevents silibinin-induced DNA double-strand breaks and glioma cell death (130). Growing evidence suggests that silibinin-induced cell death is likely to be associated with ferroptosis, but more studies are needed to prove it.

### 4.3 Radiotherapy

Radiotherapy is a highly effective and targeted treatment for cancers (181). The main molecular target of ionizing radiation (IR) is DNA, leading to a whole range of DNA damage, including double-strand breaks, cross-links and complex chromosomal rearrangements (187). It also leads to an increase in intracellular ROS by eliciting radiolysis of water (187, 188). IR induces apoptosis, senescence, methuosis and other cellular outcomes (189). IR-induced DNA damage is initially recognized by ataxia telangiectasia mutated (ATM), and after a complex signaling cascade, this damage may eventually be corrected by DNA repair mechanisms (190, 191). Tumor cells also inhibit apoptosis, and these mechanisms also contribute to radiotherapy resistance (134). Sensitizing cancer cells to radiation through alternative cell death pathways (such as ferroptosis) is a promising way to improve radiotherapy outcomes.

It was found that the antitumor efficacy of radiation may be driven by triggering ferroptosis in some contexts, and that ferroptosis inducers may effectively lead to radiosensitization (134). Iron-containing water prior to radiotherapy has been proven to stimulate glioma cell death through apoptosis and ferroptosis, thereby increasing treatment efficiency (192). In U87 cell line of glioma, synergistic effects of erastin and RSL3 with radiation promote clonogenic ferroptosis (134). A recent study found that IR promotes the expression of ACSL4 in addition to inducing ROS, suggesting a strong induction of ferroptosis by IR. The researchers suggested that IR also induces adaptive responses involving SLC7A11 or GPX4 induction to promote tumor cell survival during radiotherapy, which is one of the reasons for radioresistance. Inhibition of SLC7A11 or GPX4 induces resensitization of radiation-resistant cancer cells to IR-induced ferroptosis, leading to radiosensitization (193). The specific mechanisms of these alterations need to be explored. Further, the effect of *in vivo* radiation therapy is thought to be dependent on the presence of CD8^+^ T cells, and Wang et al. found that CD8^+^ T cells regulate tumor ferroptosis *via* IFNγ (194, 195).

### 4.4 Immunotherapy

Current immunotherapy trials in glioma are focused on immune checkpoint inhibitors, vaccines designed to induce immune responses by increasing the recruitment of antigen-specific effector T cells to tumor sites, chimeric antigen receptor (CAR)-T cells, and oncolytic viruses (196). These approaches have had some achievements, although there are currently many obstacles to immunotherapy in glioma. In GBM, the upregulation of immunosuppressive factors and recruitment of Treg cells can be detected after CART-EGFRvIII infusion (197). This suggests that multiple immune escape mechanisms in GBM are challenging to overcome. We consider that identifying the intersections of immunotherapy and ferroptosis in glioma may be expected to improve therapeutic effectiveness.

Some association exists between immunotherapy and ferroptosis. IFNγ derived from CD8^+^ T cells activated by immunotherapy and ATM activated by radiotherapy synergistically inhibit SLC7A11, inducing ferroptosis in tumor cells (194). Recent studies have shown that the ferroptosis suppressors CD44, HSPB1, and SLC40A1 are significantly associated with prognosis in GBM and correlated with immunosuppression. Acetaminophen might have an antitumor function in GBM by regulating CD44, HSPB1, and SLC40A1 to induce ferroptosis (198). Since most tumors including glioma are much more immunoreactive to TfR1 than normal brain tissue, this component may have the necessary properties to be a target for brain tumor immunotherapy (23). In glioma microenvironment, enhanced ferroptosis was shown to induce immune cell activation and infiltration, but weakened anti-tumor cytotoxic killing (199).

### 4.5 Nanotherapy

Technological advances promote new nanoscale diagnostic and therapeutic approaches in cancer medicine. We have mentioned above that the BBB is one of the reasons for resistance to drug treatment such as chemotherapy, while nanomaterials rely on their favorable physicochemical properties to be excellent transport vehicles capable of crossing the BBB (200). Several methods using nanocarriers, such as liposomes, micelles, metal ions, and nanoparticles, have been investigated for intracerebral drug delivery (201–203). Nanotechnology can be used to improve direct local treatment of glioma by extending the half-life of encapsulated drugs or providing a sustained release system (201). Nanotechnology may improve the efficacy of ferroptosis inducers, which is expected to develop a promising new approach for the treatment of glioma.

Gold nanocages (AuNCs) as carriers loaded with doxorubicin (DOX) and L-buthionine sulfoximine (BSO) evoke ferroptosis and immune responses in cancer therapy. DOX increases ROS levels, BSO restrains GSH levels, and the ROS production is further amplified by the photothermal effect mediated by AuNCs under laser irradiation (204). In glioma therapy, a recent paper reported a new nanoparticle-mediated drug delivery system that patches heparin-based nanoparticles loaded with DOX to the surface of natural grapefruit extracellular vesicles (205), however, it didn’t mention whether this system could trigger ferroptosis in glioma cells. Nevertheless, the design and successful application of these nanoparticle-dependent delivery systems show us the prospect of nanotechnology in the delivery of ferroptosis inducers for the therapy of glioma.

## 5 Conclusions and perspectives

In this review, we discuss the mechanisms and metabolic features associated with ferroptosis in glioma, and summarize other advances including regulatory targets and pathways and the intersections between ferroptosis and different forms of programmed cell death. We also provide perspectives on the application of ferroptosis in different therapeutic modalities. However, ferroptosis, as a new form of cell death, has not been extensively studied in glioma.

Many questions remain to be explored in depth. Ferroptosis is iron-dependent, iron can be involved in the Fenton reaction and also act as a cofactor to promote oxidative enzymes to engage in reactive oxygen species generation, but the role of executive factors downstream of lipid oxidation has not been elucidated by current studies (206). Moreover, what other genes and metabolic processes are involved in the occurrence of ferroptosis in glioma cells, and if there are other key regulators? Although we have concluded the crosstalk between ferroptosis and other forms of death in glioma, more discoveries are needed to address the question of how to combine different types of cell death with ferroptosis to improve killing of cancer cells.

Several studies show a remarkable potential of ferroptosis in eliminating aggressive malignancies resistant to traditional therapies. Erastin, a ferroptosis inducer, has been used to sensitize GBM cells to TMZ by blocking SLC7A11 and reducing cystathionine-γ-lyase activity. It has been shown that certain human gliomas may be sensitive to the combination therapy of a ferroptosis inducer and radiation (134). Furthermore, how to target ferroptosis induction to eliminate drug-resistant glioma cells while minimizing the impact on normal tissues is a critical therapeutic issue. And the treatment of CNS tumors such as glioma differs from other tumors, the use of ferroptosis inducers requires consideration of BBB penetration for optimal drug concentrations. Possibly nanotherapy be an ideal approach, with the development of nanomaterial technology, the combination inducing ferroptosis with nanotechnology enhances the stability, biosecurity, targeting, and controlled release of drugs to glioma cells. Research on ferroptosis provides new biomarkers and prospective targets for glioma treatment, the potential clinical application remains to be further investigated. In the future, many issues need to be clarified in more epigenetic molecules and detailed mechanistic insights to design the effective cancer therapy strategies based on ferroptosis.

## Author contributions

CQ is the corresponding author and organized this review. JS, NY, and MH analyzed the articles and drafted the manuscript. All authors contributed to the article and approved the submitted version.

## Funding

This work was supported by the Natural Science Foundation of China (81803045, 82103277), the China Postdoctoral Science Foundation (2020M672072), the Research Project of Jinan Microecological Biomedicine Shandong Laboratory (JNL-2022041C), Shandong Excellent Young Scientists Fund Program (2022HWYQ-035), Shandong Provincial Natural Science Foundation (ZR2021QH030), and the Qilu Young Scholar Program of Shandong University, China.

## Conflict of interest

The authors declare that the research was conducted in the absence of any commercial or financial relationships that could be construed as a potential conflict of interest.

## Publisher’s note

All claims expressed in this article are solely those of the authors and do not necessarily represent those of their affiliated organizations, or those of the publisher, the editors and the reviewers. Any product that may be evaluated in this article, or claim that may be made by its manufacturer, is not guaranteed or endorsed by the publisher.
